# The Role of Outer Membrane Protein(s) Harboring SLH/OprB-Domains in Extracellular Vesicles’ Production in *Synechocystis* sp. PCC 6803

**DOI:** 10.3390/plants10122757

**Published:** 2021-12-14

**Authors:** Delfim Cardoso, Steeve Lima, Jorge Matinha-Cardoso, Paula Tamagnini, Paulo Oliveira

**Affiliations:** 1 MABBS—Mestrado em Aplicações em Biotecnologia e Biologia Sintética, Faculdade de Ciências, Universidade do Porto, R. Campo Alegre s/n, 4169-007 Porto, Portugal; delfim.cardoso@i3S.up.pt; 2 i3S—Instituto de Investigação e Inovação em Saúde, Universidade do Porto, R. Alfredo Allen 208, 4200-135 Porto, Portugal; steeve.lima@i3S.up.pt (S.L.); jorge.cardoso@i3S.up.pt (J.M.-C.); pmtamagn@ibmc.up.pt (P.T.); 3 IBMC—Instituto de Biologia Molecular e Celular, Universidade do Porto, R. Alfredo Allen 208, 4200-135 Porto, Portugal; 4 MCbiology Doctoral Program, ICBAS—Instituto de Ciências Biomédicas Abel Salazar, Universidade do Porto, R. Jorge de Viterbo Ferreira 228, 4050-313 Porto, Portugal; 5 Departamento de Biologia, Faculdade de Ciências, Universidade do Porto, R. Campo Alegre s/n, 4169-007 Porto, Portugal

**Keywords:** cyanobacteria, SLH-protein domain, OprB-protein domain, outer membrane proteins, extracellular vesicles, iron uptake

## Abstract

Cyanobacteria are a group of photosynthetic prokaryotes that contribute to primary production on a global scale. These microorganisms release vesicles to the extracellular environment, spherical nanosized structures, derived essentially from the outer membrane. Even though earlier works in model Gram-negative bacteria have hypothesized that outer membrane stability is crucial in vesicle formation, the mechanisms determining vesicle biogenesis in cyanobacteria remain unknown. Here, we report on the identification of six candidate genes encoding outer membrane proteins harboring SLH/OprB-domains in the genome of the model cyanobacterium *Synechocystis* sp. PCC 6803. Using a genetics-based approach, one gene was found to encode an essential protein (Slr1841), while the remaining five are not essential for growth under standard conditions. Vesicle production was monitored, and it was found that a mutant in the gene encoding the second most abundant SLH/OprB protein in *Synechocystis* sp. PCC 6803 outer membrane (Slr1908) produces more vesicles than any of the other tested strains. Moreover, the Slr1908-protein was also found to be important for iron uptake. Altogether, our results suggest that proteins containing the SLH/OprB-domains may have dual biological role, related to micronutrient uptake and to outer membrane stability, which, together or alone, seem to be involved in cyanobacterial vesicle biogenesis.

## 1. Introduction

Extracellular vesicles (EVs) are nanosized structures (20–400 nm in diameter) presumably released by the cells of all organisms [1,2,3]. Despite their reduced size, EVs are rich in content, as they are composed of different biomolecules, including lipids, proteins, nucleic acids, and metabolites [4,5]. Delimited by a lipid bilayer, and without the capacity of replicating, EVs are not just random fragments of the cell membrane/wall, as it has been repetitively shown that their biochemical composition can be the product of selective packaging e.g., [6,7]. In agreement, EVs have been implicated with a plethora of biological roles, ranging from secretion of target molecules to inter-species and even inter-kingdom communication [4,5,8]. Remarkably, bacterial EVs have been proposed to be at the very origin of the eukaryotic endomembrane system [9]; most endosymbiotic theories postulate that eukaryotic cells must have originated from an archaeal host that engulfed an alphaproteobacterium (the mitochondrial ancestor) [10]. However, it remains highly debatable as to how the eukaryotic endomembrane system appeared, with such an elaborate vesicle trafficking organization. According to Gould et al. [9], the mitochondrial ancestor naturally released EVs, which, confined within the host’s cytosol, accumulated and fused, either with each other or with the host’s plasma membrane, giving rise to the primordial endomembrane system. In the particular case of green algae and plants, a better understanding of the origin, dynamics and response capacity of its cellular endomembrane system will require a deeper look into the evolutionary ancestors of chloroplasts, cyanobacteria.

The Cyanobacteria phylum is a large group of the domain Bacteria, including species living in, e.g., marine, freshwater or terrestrial environments, both as free-living organisms or in symbiotic associations. These photoautotrophic microorganisms play a key role in the biogeochemical cycle of carbon on a global scale. Estimates predict a global net primary production of 12 Gigatonnes each year for marine cyanobacteria only, which represents approximately 25% of ocean net primary production [11]. The first comprehensive study documenting the capacity of cyanobacteria to release EVs became available in 2014 [12]. In that study, cyanobacterial EVs were proposed to function as vehicles for the movement of carbon through marine food webs, as vectors for horizontal gene transfer, and as decoys for predators and phages [12]. After that work, others followed, mainly reporting the capacity of other cyanobacterial strains to vesiculate, leaving many aspects regarding cyanobacterial EVs biology unaddressed. One topic that has not been studied relates to how vesicles are formed and released in these microorganisms. In well-studied Gram-negative bacteria, such as *Escherichia coli* or *Pseudomonas aeruginosa*, several models have been proposed to explain the processes of EVs biogenesis, most of them positing that outer membrane (OM) stability is a fundamental aspect in EVs formation [4,5,8]. Related to such hypothesis, some players have been identified, such as different types of OM proteins, including the Lpp, or porins of the OmpA-family. However, these proteins are not universal, as not all Gram-negative bacteria harbor genes encoding those players. One such example is the Lpp protein, which is absent in cyanobacteria [8]. In fact, the cell wall of cyanobacteria is well known for its unique features, e.g., peptidoglycan thickness and extent of its crosslinking; structural differences in the lipid A moiety of lipopolysaccharides (LPS); and unique lipid composition (mainly composed of glycolipids, like chloroplasts, instead of the ordinary phospholipids present in bacterial membranes) [13], which anticipates that cyanobacterial EVs may have rather specific formation mechanisms [8].

The goal of this work was therefore to identify OM protein candidates that could play a role in EVs formation in cyanobacteria, using the unicellular cyanobacterium *Synechocystis* sp. PCC 6803 (hereafter *Synechocystis*) as a model organism. Given the available knowledge on the OM proteome composition in *Synechocystis* [14,15,16], particular attention was paid to porins harboring the SLH (S-layer homology) and the OprB (carbohydrate-selective porin OprB) protein domains. While the SLH-domain in cyanobacterial OM porins was shown to bind the peptidoglycan layer [15], and so, could contribute to OM stability, the OprB-domain is predicted to assume a β-barrel structure, forming the porin in the OM [15,17]. Here, a combination of mutant generation, strains’ growth analyses, identification of proteomic adaptations in response to target gene disruption, and isolation and characterization of vesicles, was carried out to give a comprehensive view of the investigated mechanisms. Results show that one of the highly abundant OM proteins (Slr1908) may have dual biological role, as the respective gene-disrupted mutant strain shows an unbalanced intracellular iron content, and produces more vesicles than the wild-type. This work highlights that the study of players involved in vesicle biogenesis in cyanobacteria must be accompanied by a broader evaluation of cyanobacterial-adaptation responses.

## 2. Results and Discussion

### 2.1. The Genome of Synechocystis Harbors Six Genes Putatively Encoding SLH/OprB-Domains Containing Proteins

**Figure 1:**
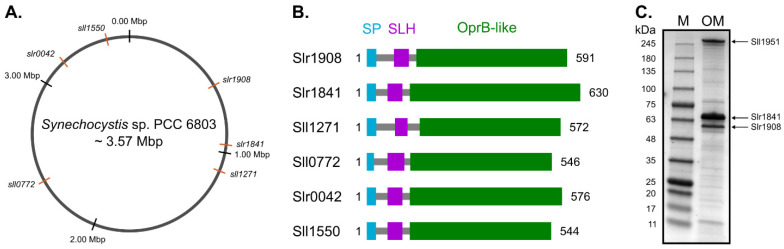
Distribution of the genes and outer membrane proteins containing S-layer homology (SLH) and carbohydrate-selective porin OprB (OprB)-domains in *Synechocystis* sp. PCC 6803. (**A**) Schematic representation of the *Synechocystis* genome, and relative position of the six genes encoding putative outer membrane (OM) proteins containing the SLH- and the OprB-domains. (**B**) Details of the size (in amino acids), and relative position of signal peptide (SP) and conserved SLH and OprB domains in the identified putative OM proteins. (**C**) PageBlue^TM^ (Thermo Fisher Scientific, Waltham, MA, USA) stained gradient 4–15% (*w/v*) SDS-polyacrylamide gel showing the protein composition of isolated *Synechocystis* OM preparations. Highlighted peptide bands were identified by mass spectrometry. Sll1951: main protein constituent of the *Synechocystis* S-layer; Slr1841 and Slr1908: SLH- and OprB-containing outer membrane proteins.

This work began with the search in the genome of *Synechocystis* for genes that could putatively encode OM proteins containing simultaneously the SLH (PF00395; IPR001119) and the OprB (PF04966; IPR007049) protein domains. The in silico analysis revealed six genes meeting these criteria—*slr1908*, *slr1841*, *sll1271*, *sll0772*, *slr0042* and *sll1550*—whose protein products show E-values lower than 4.2 × 10^−^^8^ and 3.6 × 10^−49^ for the SLH and OprB domains, respectively. In Figure 1, a schematic representation of the *Synechocystis* genome and the relative position of each gene is shown (Figure 1A). The identified candidates are not simply duplicated genes, as the predicted encoded proteins have differences in size and domain length (Figure 1B). Next, we searched the literature to understand whether transcripts of the identified genes had already been detected in high-throughput RNA experiments. To that end, we looked particularly into the work of Kopf et al. [18], in which a comparative analysis of the primary transcriptome of *Synechocystis* under 10 different growth conditions is described. While transcripts of genes *slr1908*, *slr1841*, and *slr0042* have been detected in relatively high amounts (number of reads for the respective transcriptional units were, in average, above 22,000 reads), genes *sll1271* and *sll1550* showed low transcript abundance, and transcripts of gene *sll0772* could not be detected for any of the tested conditions [18] (Supplementary Material Table S1).

We then proceeded by isolating OM fractions from *Synechocystis* wild-type cells, and separated the OM proteins by electrophoresis on SDS-polyacrylamide gels (Figure 1C). Identification by mass spectrometry of three of the most abundant proteins in the isolated OM fraction correspond to proteins Sll1951 (the main protein constituent of the *Synechocystis* S-layer), and the OM proteins Slr1908 and Slr1841. This result is in agreement with the relative abundance of transcripts of each gene (Supplementary Material Table S1), as well as with previous OM protein analyses [15,16]. Altogether, the differences detected between the identified candidates in terms of gene expression, amino acid length and composition, and protein abundance in isolated OM preparations, suggest that they may fulfill different biological functions.

### 2.2. slr1841 Encodes an Essential Protein, While the Remaining SLH/OprB-Domain Containing Outer Membrane Proteins Are Not Essential for Growth

**Figure 2:**
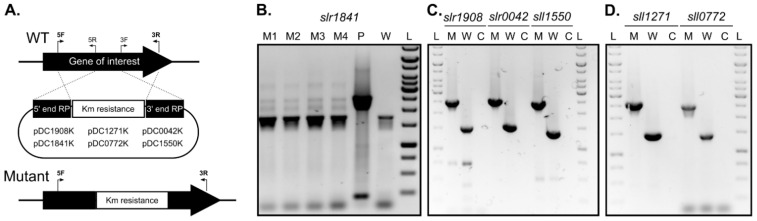
Generation of disruption mutants in genes encoding SLH-OprB-domain outer membrane proteins in *Synechocystis* sp. PCC 6803. (**A**) Scheme representing the double homologous recombination events (dashed lines) necessary to disrupt each gene of interest in the genome of *Synechocystis* wild-type (WT). The resulting mutant strain (Mutant), in which part of the gene’s coding sequence was replaced by the insertion of a kanamycin resistance cassette is also shown. Relative position of the oligonucleotides (5F, 5R; and 3F, 3R), used for amplification of the 5′ and 3′ end recombination platforms (RP), respectively, is presented. Identity of the plasmids constructed in this work and used for mutant generation is displayed. (**B**) to (**D**) Photographs of DNA-stained agarose gels showing PCR analyses of chromosome segregation in *Synechocystis* transformed with plasmids pDC1841K (for disrupting gene *slr1841*—(**B**)), pDC1908K, pDC0042K, pDC1550K (for genes *slr1908*, *slr0042*, and *sll1550*—(**C**)), and pDC1271K, pDC0772K (for genes *sll1271* and *sll0772*—(**D**)). Oligonucleotides 5F and 3R (see section (**A**)) were used for amplification, using the following templates: M1-4, 4 independent mutant colonies transformed with pDC1841K; P, plasmid DNA of pDC1841K; W, genomic DNA of *Synechocystis* wild-type; M, genomic DNA from a clone of the respective disruption mutant; C, water (negative control). L, GeneRuler 1 kb DNA Ladder (Thermo Fisher Scientific).

To assess the role of each candidate gene identified in this work and its protein product on the formation and release of EVs in *Synechocystis*, a genetics-based approach was undertaken. Constructs to generate single-gene disruption mutants by double homologous recombination of each of the identified gene candidates were prepared (Figure 2A), and *Synechocystis* cells were naturally transformed. Despite several attempts, disruption of gene *slr1841* could not be achieved. On one hand, transformation of *Synechocystis* with plasmid pDC1841K resulted in less transformants as compared to those with the other plasmids constructed in this work (data not shown). On the other hand, cells transformed with pDC1841K showed no sign of complete chromosome segregation even after several rounds of streaking onto BG11 plates with increasing antibiotic concentration (Figure 2B). Our results are in agreement with the work of Qiu et al. [19], in which the authors report to have failed in generating a full knockout mutant of gene *slr1841*. This suggests that Slr1841 likely plays an essential role in *Synechocystis* under the tested conditions, possibly involved in the uptake of micronutrients and/or in stabilizing the OM. Given the difficulty in obtaining a *Synechocystis* mutant strain lacking a functional Slr1841 protein, we therefore decided not to proceed with further studies of this gene/protein.

In contrast, the remaining five genes could be successfully disrupted, as determined by complete chromosome segregation (Figure 2C,D). Growth phenotype characterization under photoautotrophic conditions in liquid medium indicated that none of the disrupted genes encode essential proteins for *Synechocystis*, at least under standard growth conditions, as all mutant strains showed similar growth rates as compared to that of the wild-type strain (Supplementary Material Figure S1). Qiu et al. were also capable of generating full knockout mutants of genes *sll1271*, *sll0772*, *slr0042* and *sll1550*, but not of *slr1908* [19]. This discrepancy could be related to differences in *Synechocystis* sp. PCC 6803 sub-strains used in the two different studies, and/or to the approach adopted for mutant generation (complete gene deletion in [19] vs. gene disruption by partial gene deletion in the present work).

### 2.3. Disruption of slr1908 Results in Adjustments in the Outer Membrane and Extracellular Protein Composition Related to Iron Uptake

**Figure 3:**
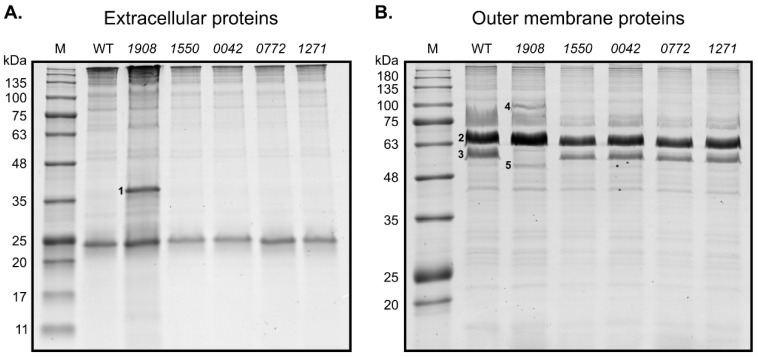
Analyses of the *Synechocystis* sp. PCC 6803 disruption mutants’ cell-free extracellular medium and outer membrane protein composition. (**A**) Protein profile of the concentrated cell-free extracellular medium from the *Synechocystis* wild-type (WT) and mutant strains (*1908*, *1550*, *0042*, *0772*, *1271*). Proteins were stained with PageBlue™ Staining Solution after separation in a gradient 4–15% (*w/v*) SDS-polyacrylamide gel. Sample loading is normalized to each culture cell density (OD_730_), volume of cell-free medium concentrated and concentration factor. (**B**) PageBlue^TM^ stained 12% (*w/v*) SDS-polyacrylamide gel showing the protein composition of isolated wild-type and mutant strains’ OM preparations. Proteins identified by mass spectrometry: 1, Slr0513 (FutA2); 2, Slr1841; 3, Slr1908; 4, Sll1406 (FhuA); 5, Sll1550; M, NZYColour Protein Marker II (NZYTech).

To determine whether disruption of SLH/OprB-protein encoding genes could result in specific proteomic adaptations in *Synechocystis*, exoprotein and OM protein fractions were isolated and analyzed by SDS-polyacrylamide gel electrophoresis (Figure 3).

Regarding the exoproteome analysis, no significant differences could be observed in peptide profile between the wild-type and the different mutant strains, except for strain *1908* (*slr1908*-disruption mutant) (Figure 3A). In this mutant, over accumulation of a single peptide with approximately 38 kDa could be observed. Mass spectrometry analysis of the respective band indicated that it corresponds to protein Slr0513.

As to the OM protein composition (Figure 3B), more differences could be detected. In mutant *1908*, the peptide band corresponding to Slr1908 is clearly absent, in agreement with the genetic modification introduced. In addition, two peptide bands showed increased accumulation as compared to the other tested strains; while the band of approximately 95 kDa was identified as protein Sll1406, the band of approximately 55 kDa was identified as the SLH/OprB-protein Sll1550. In line with the identification of Sll1550 in the OM preparation of the *slr1908*-mutant, it is possible to observe that the corresponding band in mutant *1550* is absent. Besides these differences, no further OM protein alterations could be detected in the remaining mutant strains. The reason for the lack of missing bands in OM preparations that could correspond to proteins Slr0042, Sll0772 and Sll1271 in the respective mutant strains could be related to their low abundance, and/or to the fact that the respective peptide bands separate together with other highly abundant OM proteins.

We then focused our attention on the differences observed in the exoproteome and OM protein composition of mutant *1908*. Proteins Sll1406 and Slr0513 are known iron import-related proteins. Sll1406 is homologous to the FhuA protein from *E. coli* [20]. FhuA is one of the TonB-dependent transporters (TBDTs) localized in the OM of Gram-negative bacteria [20,21]. TBDTs mediate substrate uptake in an energy-dependent mode [21], mainly iron siderophores, but also vitamins and carbohydrates [21]. It has been demonstrated that the Sll1406 protein from *Synechocystis* is a TonB-dependent transporter, together with three additional proteins [22]. These TBDTs were suggested to have redundant functions, as single knockout mutants of each of them resulted in strains lacking any distinguishable phenotype [22]. However, a quadruple knockout mutant of all TBDTs showed a sharp iron depleted phenotype, as the strain was almost completely unable to import iron siderophores, and showed strong impairment in inorganic iron uptake [22]. Moreover, Slr0513 is annotated as the iron uptake protein A2 (FutA2), a component of the Fut iron uptake system. This system is composed by two periplasmic binding proteins (FutA1 and FutA2), and two inner membrane proteins (FutB and FutC) [20]. The periplasmic proteins are iron binding proteins that mediate iron transport to the cytoplasm through the inner membrane protein complex [20,23]. In *Synechocystis*, only the FutA2 protein was found in the periplasmic space, where it is the most abundant protein therein, while the FutA1 protein was detected neither in the soluble protein fraction of the periplasm nor in the cytoplasmic membrane [24,25].

Differential accumulation of proteins in the OM (FhuA and Sll1550) and in the extracellular medium (FutA2) strongly indicates that iron uptake systems have been adapted to adjust for the absence of Slr1908. Higher abundance of FhuA in the OM can result from an attempt to increase iron uptake by other routes, namely by the TonB-dependent transport system. In addition, FutA2 accumulation in the extracellular medium may represent a strategy for iron scavenging from the medium, which would require further FutA2 internalization. Moreover, Sll1550 may also represent an additional mechanism for iron uptake; in fact, several reports show that *sll1550* gene expression is upregulated when *Synechocystis* cells are exposed to iron-limiting conditions [18,26]. Overall, the finding of increased abundance in iron uptake proteins in the *slr1908*-mutant is in agreement with the potential role proposed for the Slr1908 protein to work as a porin [16]. Kowata et al. showed that Slr1841, Slr1908 and Slr0042 are responsible for uptaking approximately 80% of inorganic ions [16]. Moreover, Slr1908 has also been suggested to work as an iron-selective porin [19]. Based on our and others’ results, the *slr1908*-mutant strain was selected for further studies to uncover its altered iron uptake phenotype, and to better understand the function of the Slr1908 protein in *Synechocystis*.

### 2.4. Mutant Strain 1908 Shows Significantly Lower Intracellular Iron Content

**Figure 4:**
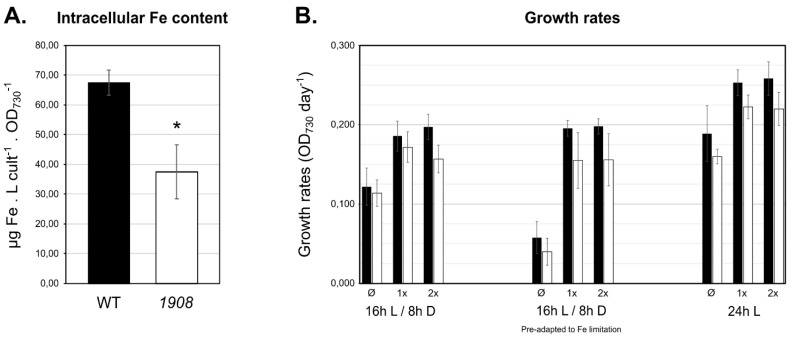
Analysis of the *Synechocystis* sp. PCC 6803 *slr1908*-mutant strain intracellular iron content and growth in medium containing different amounts of iron. (**A**) Intracellular iron concentration determined by atomic absorption spectrometry in *Synechocystis* wild-type (WT) and *slr1908*-mutant (*1908*) strains cultivated under standard liquid BG11 conditions, shown in µg of Fe. L culture^−^^1^. OD_730_^−1^. Error bars represent the standard deviations of three independent biological replicates. *, *p* < 0.05. (**B**) *Synechocystis* wild-type and *slr1908*-mutant growth rates in BG11 medium with modified iron ammonium citrate concentration, under different cultivation setups. Cyanobacterial cells were cultivated in 96-well clear bottomed microplates at 30 °C for four days. Standard BG11 medium was used as control condition (1×), or in BG11 either lacking iron ammonium citrate (Ø) or in BG11 medium containing two-fold excess iron ammonium citrate (2×). Different cultivation setups were tested: (1) cells were grown under a 16 h light (50 μmol photons m^−2^ s^−1^)/ 8 h dark cycle regime (16 h L/8 h D); (2) cells were previously pre-adapted for seven days in BG11 medium without iron ammonium citrate, and then used as inoculum for the cultivation assay, under a 16 h light (50 μmol photons m^−2^ s^−1^)/ 8 h dark cycle regime (16 h L/8 h D (pre-adapted to Fe limitation)); (3) cells grown under constant light (24 h L). Error bars represent the standard deviation of at least three independent biological replicates.

To evaluate the involvement of Slr1908 in iron uptake, the total intracellular iron content of *Synechocystis* wild-type and *slr1908*-mutant cells cultivated under standard growth conditions was investigated. To that end, cell extracts were obtained and analyzed by atomic absorption spectrometry, which determined that *slr1908*-mutant cells have approximately 40% less iron content than wild-type cells (Figure 4A), further supporting that Slr1908 is involved in iron import in *Synechocystis*. As no difference in growth could be observed between the *Synechocystis* wild-type and *1908* cells under standard conditions (Supplementary Material Figure S1), we then decided to evaluate the role of the protein under conditions with different iron availability and light regimes (Figure 4B). The decision of combining these two environmental conditions was based on the fact that different enzymatic complexes involved in the primary metabolism of *Synechocystis* (e.g., light-dependent photosystems I and II, and cytochrome b_6_f, but also terminal oxidases in respiration) require iron as a co-factor.

Overall, growth rates’ analysis showed that cyanobacterial strains cultivated under continuous light grew faster than those in light/dark cycles, as cyanobacterial growth is closely dependent on light availability [27]. Moreover, it was also possible to observe that cells cultivated in medium lacking iron ammonium citrate had lower growth rates as to those cultivated in complete medium or supplemented with extra amounts of iron ammonium citrate, in agreement with previous reports [19]. Nevertheless, irrespective of the condition analyzed, strain *1908* displayed a consistently lower growth rate when compared to the wild-type, but the detected differences were never significant (*p* > 0.05), with at least three independent biological replicates. Altogether, these results indicate that under physiological conditions combining the light regime and iron availability, absence of Slr1908 does not induce significant growth impairment, even when the intracellular iron content is reduced by 40% (Figure 4A). These results are somewhat different to those reported earlier by Qiu et al., who showed that downregulation of *slr1908* gene expression results in significantly lower growth [19]. However, the work was not carried out on a *slr1908* disruption mutant as described here, but rather on a strain in which *slr1908* gene expression was under the control of a copper inducible promoter (P*_petE_*) [19]. In such conditions, *slr1908* was transcribed in a medium containing copper, while strong repression of gene expression was achieved by removing copper from the medium. Therefore, even though Qiu et al. observe significant impairment in iron uptake in a *slr1908*-knocked-down strain, followed by an impact in its growth, this could result from synergistic effects between altered iron uptake and induced copper low availability, which could generate an effect in, e.g., electron transport in photosynthesis [19]. It is also possible that absence of a functional Slr1908 does indeed result in significant alterations in growth, but the difference is likely so small that more biological replicates would be required to validate such phenotypes. Nevertheless, the results from both works complement each other in supporting the possibility of Slr1908 in working as an iron-selective import porin.

### 2.5. Disruption of slr1908 Results in Hypervesiculation

**Figure 5:**
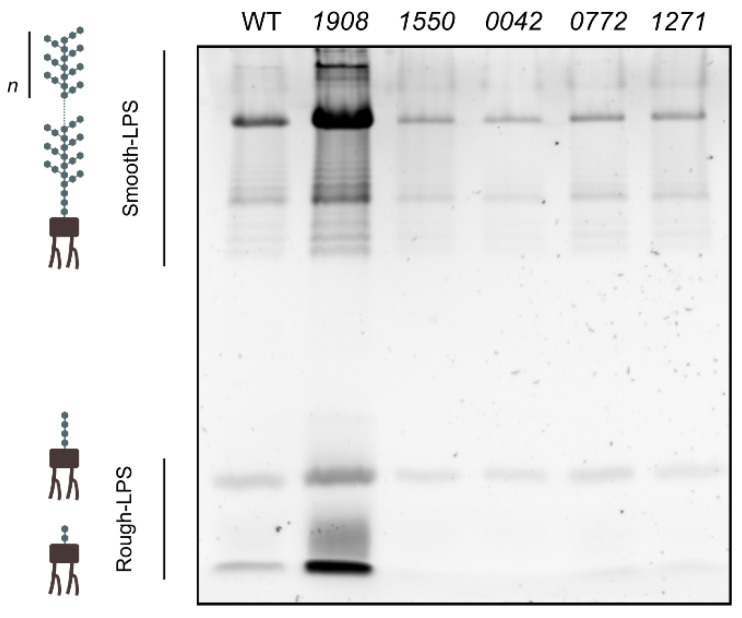
Analysis of the lipopolysaccharides (LPS) content in *Synechocystis* sp. PCC 6803 wild-type and mutant strains in cell-free extracellular medium samples. *Synechocystis* wild-type (WT) and mutant (*1908*, *1550*, *0042*, *0772*, *1271*) strains were cultivated under standard conditions, and the growth medium was collected and concentrated after cultivation. Cell-free, concentrated samples were separated on 16% (*w/v*) SDS-polyacrylamide gels and stained with Pro-Q™ Emerald 300 Lipopolysaccharide Gel Stain Kit (Thermo Fisher Scientific). Sample loading is normalized to each culture cell density (OD_730_), volume of cell-free medium concentrated and concentration factor. On the left-hand side, schematic representation of the smooth-LPS (lipid A, oligosaccharide core, and *O*-antigen), and rough-LPS (lipid A and oligosaccharide core) that are found in *Synechocystis* OM and vesicles.

The main goal of this work was to investigate the possible role of SLH-OprB OM proteins on the processes of EVs formation. As LPS are structural components of the cyanobacterial OM, their abundance in the extracellular medium was also evaluated, as a proxy for assessing possible differential vesiculation capacity between the different cyanobacterial strains, as previously determined [28,29]. Cell-free, concentrated extracellular medium samples isolated from *Synechocystis* wild-type and mutant strains were separated by electrophoresis on SDS-polyacrylamide gels, and further stained with an LPS specific protocol (Figure 5).

**Figure 6:**
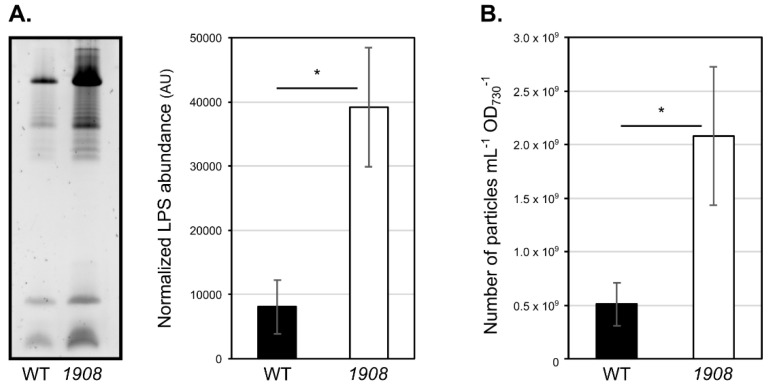
Vesiculation capacity of *Synechocystis* sp. PCC 6803 wild-type and *slr1908*-mutant strains. Cyanobacterial strains were cultivated under standard conditions and their extracellular vesicles (EVs) isolated by ultrafiltration and ultracentrifugation. (**A**) EVs samples from wild-type (WT) and *slr1908*-mutant were separated by electrophoresis on 16% (*w/v*) SDS-polyacrylamide gel and stained with Pro-Q™ Emerald 300 Lipopolysaccharide Gel Stain (Thermo Fisher Scientific). Sample loading is normalized to each culture cell density (OD_730_), volume of cell-free medium concentrated and concentration factor. LPS band intensity was quantified by densitometry using the ImageJ software, resulting in the graph shown onto the right-hand side. (**B**) Particle concentration determination of the isolated EVs samples by nanoparticle tracking analysis. Error bars represent the standard deviation of three independent biological replicates. *, *p* < 0.05.

Mutant *1908* was the only strain showing a significant increase in the total amount of extracellular LPS as compared to the wild-type strain, suggesting that it releases more EVs. To verify this result, we then proceeded with EVs isolation from wild-type and *1908*. The resulting samples were then characterized by evaluating LPS bands’ profile and abundance, nanoparticle tracking analysis (NTA), and transmission electron microscopy. In agreement with the analysis of complete extracellular medium concentrated samples (Figure 5), analysis of the LPS content in isolated EVs samples, followed by densitometry quantification, indicated that *1908* contains approximately five-fold more LPS then the wild-type strain (Figure 6A). Furthermore, NTA allowed for the quantification of the total amount of nano-sized particles in the samples (Figure 6B). This technique shows that *slr1908*-mutant strain releases up to four-fold more nanoparticles than the parental strain. Together, these results strongly suggest that *1908* is a hypervesiculating strain.

**Figure 7:**
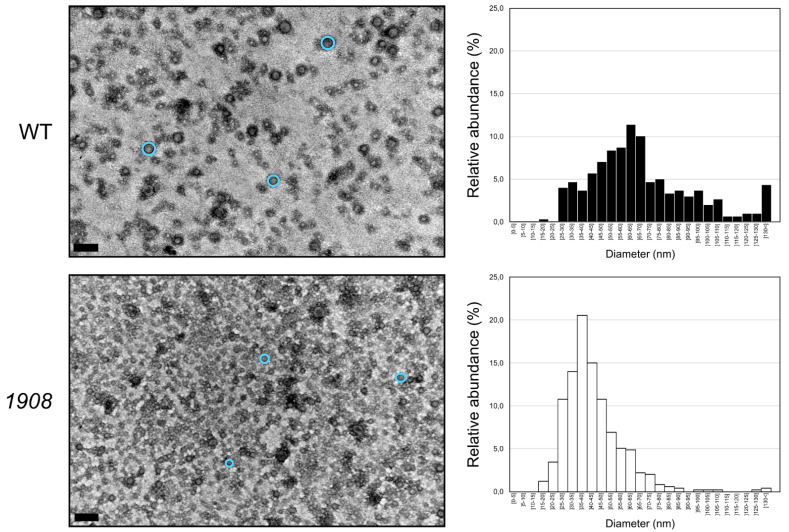
Structure and size distribution analysis of extracellular vesicles isolated from the *Synechocystis* sp. PCC 6803 wild-type and *slr1908*-mutant based on transmission electron microscopy. Cyanobacterial strains were cultivated under standard conditions, and their EVs isolated by ultrafiltration and ultracentrifugation. EVs from the *Synechocystis* wild-type (WT) and *slr1908*-mutant (*1908*) were negatively stained and visualized by transmission electron microscopy. Three selected vesicles in each micrograph are labeled as light-blue circles to indicate size and shape of representative EVs. Size bar: 200 nm. Vesicle diameter was determined by direct measurement from electron micrographs of three independent biological replicates, resulting in the histograms shown onto the right-hand side. EVs were separated in 5 nm size groups, distributed between 0 and 130 nm, and in a group comprising those with a size higher than 130 nm. Abundance of each size group is presented as relative figures, i.e., number of vesicles with a given diameter range relative to the total number of vesicles measured.

Transmission electron micrographs of negatively stained isolated EVs (Figure 7) show the accumulation of nano-sized spherical structures that resemble those of EVs reported earlier for *Synechocystis* [8,29,30] and other Gram-negative bacteria [4,5,12]. In line with LPS and NTA analyses, electron micrographs further support the higher vesiculation capacity of *1908*. In addition, this technique also allowed the detection of shifts in size of the released EVs. Wild-type EVs have a broad diameter distribution, and more than 50% of the measured vesicles range between 45 and 75 nm in diameter. In contrast, EVs produced by *1908* have a more homogeneous size distribution, with approximately 50% of the measured EVs with a diameter between 30 and 45 nm (Figure 7). However, the biological aspects accounting for the differences in EVs’ size between the wild-type and the mutant strain remain unknown.

The increase in vesicles’ release by *1908* is in line with the proposed biogenesis mechanism for Gram-negative bacteria, based on the modulation of links between the OM and the remaining cell envelope components [5,8]. This suggests that cyanobacteria can release EVs following this particular EVs production route. Nevertheless, *1908* also shows extensive adaptations to iron uptake. Therefore, it should not be discarded that mutant cells may be using EVs as an alternative iron uptake system. In this regard, it has been suggested that bacterial EVs can be loaded with proteins or other molecules to scavenge iron from the extracellular medium, and later be internalized by the cells [4,5,31]. Once the protein content from the isolated EVs was not analyzed in this work, further testing is needed to clarify the roles played by the EVs in *1908*.

## 3. Conclusions and Future Perspectives

This work shows that inactivation of *slr1908*, encoding a major OM protein harboring SLH/OprB-domains, causes a reduction in intracellular iron content in *Synechocystis*, and results in a number of protein adaptations at the outer membrane and extracellular protein levels. In addition, absence of Slr1908 leads to an increased vesicle production. We suggest that outer membrane stability in cyanobacteria is dependent on the links established by proteins harboring SLH/OprB-domains and the underlying peptidoglycan layer. By removing Slr1908, we hypothesize that outer membrane stability is compromised, leading to hypervesiculation. Nevertheless, the molecular mechanisms determining SLH-protein domain binding to the peptidoglycan layer, and the players involved in modulating those interactions, represent two of the key aspects that deserve further examination. There are indications that pyruvylation of the peptidoglycan-associated polysaccharide is essential for the binding of the SLH-protein domain [32], but the picture is far from complete in cyanobacteria. Moreover, the dance between the different major OM proteins in response to different environmental and physiological conditions is an additional topic that warrants investigation. Altogether, this work highlights that the study of mechanisms determining vesicle biogenesis in cyanobacteria must be closely combined with inorganic ions uptake, OM protein composition, and cell envelope stability, which will be essential for determining the cause–effect relationship among players with possible dual biological role.

## 4. Materials and Methods

### 4.1. Strains and Maintenance Conditions

The unicellular, non-nitrogen fixing cyanobacterium *Synechocystis* sp. PCC 6803 (non-motile and with S-layer) was routinely maintained in liquid BG11 medium [33], in Erlenmeyer flasks with agitation (70 rpm), under a light regimen of 16 h light (15 µmol photons m^−2^ s^−1^)/8 h dark, at 28 °C. For cultivation in solid plates, BG11 medium was supplemented with 1% (*w/v*) Noble agar (Difco), and 4.79 g L^−1^ of sodium thiosulfate pentahydrate. *Synechocystis* mutant strains generated in this work were cultivated similarly, except that BG11 medium was supplemented with 100 µg mL^−1^ kanamycin. For monitoring growth, optical density at 730 nm (OD_730_) was determined by spectrophotometry.

### 4.2. In Silico Identification of Genes Encoding SLH/OprB-Domain Containing Proteins

The deduced protein sequences from the genome of *Synechocystis* sp. PCC 6803 were used as a query in the web resource SMART (Simple Modular Architecture Research Tool) [34,35]. The search of proteins with an architecture combining the specific protein domains SLH (PF00395; IPR001119) and OprB (PF04966; IPR007049) was carried out. Protein candidates with an E-value lower than 4.2 × 10^−^^8^ and 3.6 × 10^−49^ for the SLH and OprB domains, respectively, were selected for further analysis.

### 4.3. Isolation and Analysis of Synechocystis Outer Membrane Proteins

*Synechocystis* cultures were first prepared to an initial OD_730_ of 0.5 in liquid BG11 medium, and cultivated in Erlenmeyer flasks with agitation (70 rpm) at 28 °C, under the same light and temperature conditions as described (see *4.1 Strains and maintenance conditions*) up to an OD_730_ of 1.0–1.5. These were then used to inoculate cultures (initial OD_730_ of 0.05) maintained in liquid BG11 medium, in glass gas washing bottles with aeration (1 L min^−1^), under a light regimen of 16 h light (40 µmol photons m^−2^ s^−1^)/8 h dark. When cultures reached an OD_730_ of approximately 1.0–1.5, OMs were isolated as described initially by [36] and adapted by [37]. In brief, cells were pelleted at 4400× *g* for 10 min at room temperature and the pellet corresponding to 50 mL of grown culture was washed in 1 mL of buffer O (10 mM Tris-HCl, pH 7.45). The mixture was then transferred to a 1.5 mL microfuge tube and centrifuged at 16,100× *g* for 2 min at 4 °C. Cells were suspended in 400 μL of buffer O and lysed by sonication (Branson Sonifier 250 (output 3–4, 50% duty cycle)) in 5 cycles of 15 sec power, 15 sec on ice. Then, cell lysates were centrifuged for 15 sec at 16,100× *g* (4 °C) to remove unbroken cells and heavy cellular debris, and the supernatant was further centrifuged for 30 min at 16,100× *g* (4 °C). The resulting pellet was washed in 200 μL of buffer O, and centrifuged once again for 30 min under the same conditions. Next, the pellet was suspended in 200 μL buffer O, and mixed with 250 μL of buffer O containing 2% (*v/v*) N-Lauroylsarcosine. The mixture was incubated for 30 min with agitation in a Roto-Torque (Cole-Parmer) at room temperature. Outer membranes were then collected by centrifugation (45 min at 16,100× *g* (4 °C)), and washed twice in 400 μL of buffer O. In the end, isolated OMs were suspended in 50 μL of buffer O and stored at −20 °C. Proteins were separated by electrophoresis either on gradient 4–15% (*w/v*) (Bio-Rad) or on homogeneous 12% (*w/v*) SDS-polyacrylamide gels. Peptide band detection was performed either with colloidal Coomassie brilliant blue (Sigma) staining or PageBlue™ Protein Staining Solution (Thermo Fisher Scientific), and pictures of the stained gels were acquired with a GS-800 calibrated imaging densitometer (Bio-Rad).

### 4.4. Protein Identification by NanoLC-MS/MS

Selected polyacrylamide gel bands were analyzed by mass spectrometry at the i3S Proteomics Unit. Gel bands were washed twice with 50% acetonitrile (ACN) in 50 mM triethylammonium bicarbonate (TEAB) with shaking at 1500 rpm for 5 min, and further treated with ACN twice. Next, proteins were reduced with 25 mM dithiothreitol (DTT) for 20 min at 56 °C and alkylated with 55 mM iodoacetamide (IAA) for 20 min at room temperature in the dark, followed by the same wash procedure. Proteins were then digested with trypsin (240 ng) in 50 mM TEAB/0.01% (*w/v*) surfactant for 60 min at 50 °C. Peptide gel extraction was performed with 2.5% trifluoroacetic acid (TFA) followed by 50% ACN, 0.1% TFA. Then, samples were dried, suspended in 10 µL 0.1% TFA and cleaned by C18 reverse phase chromatography following the manufacturer’s instructions (ZipTip, Merck, Darmstadt, Germany). Sample protein identification and quantification was performed by nano liquid chromatography mass spectrometry (nanoLC-MS/MS) with a 90 min chromatographic separation run. The specific MS parameters were: MS maximum injection time, 100 ms; dd settings: minimum AGC target 7.0 × 10^3^, intensity threshold 6.4 × 10^4^, and dynamic exclusion 20 s. Data acquisition was controlled by Tune 2.9 software (Thermo Scientific, Waltham, MA, USA). The UniProt database for the taxonomic selection *Synechocystis* sp. PCC 6803 was considered for protein identification. Protein identification was performed with the Proteome Discoverer software (v2.3.0.523, Thermo Scientific).

### 4.5. Mutant Generation

To generate mutants of the genes encoding OM porins in *Synechocystis*, a classical double homologous recombination approach was undertaken [38]. To that end, one plasmid was generated for each gene of interest. The recombination platforms (approximately 600 bp) for each gene disruption were amplified by PCR using specific oligonucleotides (see Supplementary Material Table S2), and with *Synechocystis* genomic DNA as template. As the oligonucleotides’ sequences were designed to harbor recognition sites for various restriction enzymes, the DNA fragment corresponding to the 5′ recombination platform was digested with XhoI and PstI, while the 3′ recombination platform was digested with PstI and BamHI, following the manufacturer’s instructions (Thermo Fisher Scientific). In addition, plasmid pBluescript SKII (+) was digested with XhoI and BamHI. Assembly of the three digested DNA fragments was performed by ligation with T4 DNA ligase (Thermo Fisher Scientific), and later transformed into *E. coli*. Identity of the successfully assembled plasmids was determined by Sanger sequencing (StabVida), resulting in plasmids pDC1841, pDC1908, pDC0042, pDC1550, pDC0772, and pDC1271. Later, the kanamycin resistance cassette from the plasmid pUC4K (GE Healthcare) was amplified using specific oligonucleotides (Supplementary Material Table S2). The resulting DNA fragment was digested with PstI, as well as each of the generated plasmids. The two DNA fragments were assembled by ligation and transformed into *E. coli*. Kanamycin resistant colonies were selected for plasmid isolation, and the resulting plasmids were named pDC1841K, pDC1908K, pDC0042K, pDC1550K, pDC0772K, and pDC1271K. The generated plasmids were used to naturally transform *Synechocystis* according to Heidorn et al. [38]. Transformants were transferred repeatedly over the course of 4–5 weeks to BG11 agar plates supplemented with increasing concentrations of kanamycin to ensure complete chromosome segregation.

### 4.6. Analysis of Synechocystis Wild-Type and Mutant Strains’ Growth Capacities

To monitor growth of the various *Synechocystis* strains under standard conditions, cyanobacteria were first inoculated in 100 mL Erlenmeyer flasks to an initial OD_730_ of 0.5, supplemented with kanamycin when necessary, and cultivated at 28 °C, under a light regimen of 16 h light (15 µmol photons m^−2^ s^−1^)/ 8 h dark, with agitation (70 rpm). Cultures with an OD_730_ of approximately 1.0–1.8 were then diluted to an OD_730_ of 0.4 with BG11 liquid medium, distributed onto 96-well clear bottomed microtiter plates in technical triplicates, and incubated at 30 °C with a 16 h light (50 μmol photons m^−2^ s^−1^)/ 8 h dark cycle with agitation (150 rpm). On a daily basis, cells were suspended by pipetting, and optical density at OD_730_ was monitored on a microplate reader (Synergy Mx, BioTek, Winooski, VT, USA).

To evaluate the effect of combing iron and light availability on the growth capacity of *Synechocystis* wild-type and *slr1908*-mutant strain, similar analyses were performed on 96-well clear bottomed microtiter plates. Growth was monitored in cultures cultivated in BG11 medium, or in modified BG11 medium, in which iron ammonium citrate was either absent or supplied in a two-fold amount as compared to standard BG11. Moreover, three cultivation setups were tested, including: (1) cells cultivated in constant light; (2) cells cultivated in light/dark cycles (16 h light/8 h dark); or (3) cells pre-adapted for one week in modified BG11 medium lacking iron ammonium citrate (in that period, cultivation was carried out in Erlenmeyer flasks, in a 16 h light/8 h dark cycle regimen). Regardless of the tested condition, *Synechocystis* strains were washed three times in modified BG11 medium lacking iron ammonium citrate prior to being distributed onto the microtiter plates. Optical density was monitored daily on the microplate reader Synergy Mx.

At least three independent biological replicates were performed for each strain and condition. For analysis, growth rates were calculated between the first and fourth days of cultivation, and are expressed in OD_730_.day^−1^. Statistical analyses were performed by one-way ANOVA, by comparing the growth rate of each strain in a given condition and that of the wild-type in the same growth condition.

### 4.7. Quantification of Intracellular Iron Levels by Atomic Absorption Spectrometry

The intracellular levels of iron in *Synechocystis* wild-type and *slr1908*-mutant strain were quantified by atomic absorption spectrometry. Cells were cultivated in standard conditions (BG11 medium, and in gas washing bottles, with aeration (1 L air min^−1^)) up to an OD_730_ of 0.8–1.2. Biomass was then collected by centrifugation at 4400× *g* for 10 min, and washed several times with extraction buffer (20 mM Tris-HCl, 5 mM EDTA, pH 7.6) for complete removal of BG11 traces. Later, cell pellets were mixed with 100 µL of 65% HNO_3_, and incubated at 75 °C for 12 min, vigorously vortexed every 2 min. Then, Milli-Q^®^ grade water was added, samples mildly vortexed, and centrifuged at 16,000× *g* for 5 min at room temperature. Iron levels were determined in the resulting supernatant with an atomic absorption spectrometer coupled to a flame atomization detector (Perkin Elmer). Quantifications (using three independent biological replicates for each strain) were performed against calibration curves using specific aqueous pattern solutions. Results are presented in µg of metal L^−1^ culture OD_730_^−1^, and statistical analysis was performed by one-way ANOVA.

### 4.8. Isolation, Concentration and Analysis of Cyanobacterial Extracellular Media

To analyze the exoprotein and extracellular LPS content in the growth medium, *Synechocystis* wild-type and mutant strains were cultivated as described above (see *4.3. Isolation and analysis of Synechocystis outer membrane proteins*). After cell harvesting, the extracellular medium was filtered through 0.2 µm pore-size filters, and concentrated by ultrafiltration with Amicon Ultra-15 Centrifugal Filter units (Millipore, Burlington, MA, USA) with a nominal molecular weight limit of 3 kDa [29,39]. Prior to analysis, samples were normalized by the volume of cell-free extracellular medium concentrated, concentration factor, and respective culture’s cell density (OD_730_). For exoproteome analysis, samples were separated by electrophoresis either on gradient 4–15% (*w/v*) (Bio-Rad, Hercules, CA, USA) or on homogeneous 12% (*w/v*) SDS-polyacrylamide gels. Peptide band detection was performed with colloidal Coomassie brilliant blue (Sigma-Aldrich, St. Louis, MO, USA) staining, and pictures of the stained gels were acquired with a GS-800 calibrated imaging densitometer (Bio-Rad). For detection of extracellular LPS, the protocol described by Oliveira et al. [29] was followed. In brief, samples were heat denatured either in 1× solubilization buffer (2×: 20% (*v/v*) glycerol, 10% (*v/v*) 2-mercaptoethanol, 4% (*w/v*) SDS, 0.05% (*w/v*) bromophenol blue in 125 mM Tris-HCl pH 6.8) at 95 °C for 10 min. Heat denatured samples were incubated with 0.2 U of type XIV protease from *Streptomyces griseus* (Sigma-Aldrich) at 37 °C for 30 min. Samples were then separated by electrophoresis on 16% (*w/v*) SDS-polyacrylamide gels, and LPS were stained with Pro-Q^®^ Emerald 300 Lipopolysaccharide gel staining kit (Life Technologies, Carlsbad, CA, USA). LPS were visualized on a Gel Doc^TM^ XR + UV transilluminator system (Bio-Rad), and LPS band intensity was quantified by densitometry using the ImageJ software.

### 4.9. Isolation and Characterization of Cyanobacterial Extracellular Vesicles

For isolation of cyanobacterial EVs, cells were cultivated and the extracellular medium recovered as described above. Next, the cell-free extracellular medium was concentrated by ultrafiltration with centrifugal filters (Pall, New York, NY, USA) with a nominal molecular weight limit of 100 kDa to approximately 10–20 mL. The concentrated solution was then ultracentrifuged (70 Ti fixed angle rotor, Beckman Coulter Optima L80 XP Ultracentrifuge, Brea, CA, USA) using polycarbonate bottles with cap assembly (#355618) for 3 h at 100,000× *g* at 4 °C. Finally, the resulting pellet was suspended in approximately 200 µL of sterile BG11 growth medium, and stored at −80 °C until further analysis.

To evaluate the amount and band profile of the LPS in the isolated *Synechocystis* EVs, samples were prepared, separated by electrophoresis, and gels stained as described above. In addition, EVs samples were visualized by negative-staining transmission electron microscopy mainly as described elsewhere [29]. A volume of 10 µL of sample were added on formvar/carbon film-coated mesh nickel grids (Electron Microscopy Sciences~, Hatfield, PA, USA) and left standing for 2 min. Using filter paper, the liquid in excess was removed, and 5 µL of 1% (*w/v*) uranyl acetate was used to cover the grids and left standing for 10 s, after which liquid in excess was again removed with filter paper. Observation was performed using a Jeol JEM-1400 transmission electron microscope at 80 kV. Finally, EVs were analyzed on a NanoSight NS300 NTA Dev Build 3.2.16 (Malvern, Malvern, UK), equipped with an sCMOS camera. Samples were diluted, and further analyzed as described [29]. In brief, three movies of 30 s were recorded for each sample (with a threshold of 10 to 50 particles per frame), with the following capturing settings: camera level 14–16, slider shutter 1300, slider gain 512, at 23 °C, and with a syringe pump speed of 40. Data acquisition and processing were performed using the NanoSight NS300 NTA 3.0 software, using a detection threshold of 5.

## Data Availability

Data is contained within the article or supplementary material.

## Supplementary Materials

The following are available online at https://www.mdpi.com/article/10.3390/plants10122757/s1, Table S1: Transcript levels analysis of the *Synechocystis* sp. PCC 6803 transcriptional units (TU) harboring genes encoding SLH/OprB-domains outer membrane proteins under 10 different environmental conditions, as determined by [18]; Table S2: Oligonucleotides used in this study; Figure S1: *Synechocystis* sp. PCC 6803 disruption mutants’ growth rates determined in cultures grown under standard conditions.

## References

[1] Coelho C., Casadevall A. (2019). Answers to naysayers regarding microbial extracellular vesicles. Biochem. Soc. Trans..

[2] Raposo G., Stoorvogel W. (2013). Extracellular vesicles: Exosomes, microvesicles, and friends. J. Cell Biol..

[3] Van Niel G., D’Angelo G., Raposo G. (2018). Shedding light on the cell biology of extracellular vesicles. Nat. Rev. Mol. Cell Biol..

[4] Kulp A., Kuehn M.J. (2010). Biological Functions and Biogenesis of Secreted Bacterial Outer Membrane Vesicles. Annu. Rev. Microbiol..

[5] Schwechheimer C., Kuehn M.J. (2015). Outer-membrane vesicles from Gram-negative bacteria: Biogenesis and functions. Nat. Rev. Genet..

[6] Haurat M.F., Aduse-Opoku A., Rangarajan M., Dorobantu L., Gray M., A Curtis M., Feldman M.F. (2011). Selective Sorting of Cargo Proteins into Bacterial Membrane Vesicles. J. Biol. Chem..

[7] Rakoff-Nahoum S., Coyne M.J., Comstock L.E. (2014). An Ecological Network of Polysaccharide Utilization among Human Intestinal Symbionts. Curr. Biol..

[8] Lima S., Matinha-Cardoso J., Tamagnini P., Oliveira P. (2020). Extracellular Vesicles: An Overlooked Secretion System in Cyano-bacteria. Life.

[9] Gould S.B., Garg S.G., Martin W.F. (2016). Bacterial Vesicle Secretion and the Evolutionary Origin of the Eukaryotic Endomembrane System. Trends Microbiol..

[10] Ku C., Nelson-Sathi S., Roettger M., Sousa F.L., Lockhart P.J., Bryant D., Hazkani-Covo E., McInerney J.O., Landan G., Martin W.F. (2015). Endosymbiotic origin and differential loss of eukaryotic genes. Nat. Cell Biol..

[11] Flombaum P., Gallegos J.L., Gordillo R.A., Rincón J., Zabala L.L., Jiao N., Karl D.M., Li W.K.W., Lomas M., Veneziano D. (2013). Present and future global distributions of the marine Cyanobacteria *Prochlorococcus* and *Synechococcus*. Proc. Natl. Acad. Sci. USA.

[12] Biller S.J., Schubotz F., Roggensack S.E., Thompson A.W., Summons R.E., Chisholm S.W. (2014). Bacterial Vesicles in Marine Ecosystems. Science.

[13] Hoiczyk E., Hansel A. (2000). Cyanobacterial Cell Walls: News from an Unusual Prokaryotic Envelope. J. Bacteriol..

[14] Huang F., Hedman E., Funk C., Kieselbach T., Schröder W.P., Norling B. (2004). Isolation of Outer Membrane of *Synechocystis* sp. PCC 6803 and Its Proteomic Characterization. Mol. Cell. Proteom..

[15] Kojima S., Muramoto K., Kusano T. (2016). Outer Membrane Proteins Derived from Non-cyanobacterial Lineage Cover the Pepti-doglycan of *Cyanophora paradoxa* Cyanelles and Serve as a Cyanelle Diffusion Channel. J. Biol. Chem..

[16] Kowata H., Tochigi S., Takahashi H., Kojima S. (2017). Outer Membrane Permeability of Cyanobacterium *Synechocystis* sp. Strain PCC 6803: Studies of Passive Diffusion of Small Organic Nutrients Reveal the Absence of Classical Porins and Intrinsically Low Permeability. J. Bacteriol..

[17] Schätzle H., Brouwer E.-M., Liebhart E., Stevanovic M., Schleiff A.E. (2021). Comparative Phenotypic Analysis of *Anabaena* sp. PCC 7120 Mutants of Porin-like Genes. J. Microbiol. Biotechnol..

[18] Kopf M., Klähn S., Scholz I., Matthiessen J.K., Hess W., Voß B. (2014). Comparative Analysis of the Primary Transcriptome of *Synechocystis* sp. PCC 6803. DNA Res..

[19] Qiu G., Jiang H., Lis H., Li Z., Deng B., Shang J., Sun C., Keren N., Qiu B. (2021). A unique porin meditates iron-selective transport through cyanobacterial outer membranes. Environ. Microbiol..

[20] Katoh H., Hagino N., Grossman A.R., Ogawa T. (2001). Genes Essential to Iron Transport in the Cyanobacterium *Synechocystis* sp. Strain PCC 6803. J. Bacteriol..

[21] Noinaj N., Guillier M., Barnard T.J., Buchanan S.K. (2010). TonB-Dependent Transporters: Regulation, Structure, and Function. Annu. Rev. Microbiol..

[22] Qiu G.-W., Lou W.-J., Sun C.-Y., Yang N., Li Z.-K., Li D.-L., Zang S.-S., Fu F.-X., Hutchins D.A., Jiang H.-B. (2018). Outer Membrane Iron Uptake Pathways in the Model Cyanobacterium *Synechocystis* sp. Strain PCC 6803. Appl. Environ. Microbiol..

[23] Badarau A., Firbank S.J., Waldron K., Yanagisawa S., Robinson N., Banfield M., Dennison C. (2008). FutA2 Is a Ferric Binding Protein from *Synechocystis* PCC 6803. J. Biol. Chem..

[24] Huang F., Parmryd I., Nilsson F., Persson A.L., Pakrasi H.B., Andersson B., Norling B. (2002). Proteomics of *Synechocystis* sp. Strain PCC 6803. Mol. Cell. Proteom..

[25] Tölle J., Michel K.-P., Kruip J., Kahmann U., Preisfeld A., Pistorius E.K. (2002). Localization and function of the IdiA homologue Slr1295 in the cyanobacterium *Synechocystis* sp. strain PCC 6803. Microbiology.

[26] Hernández-Prieto M.A., Semeniuk T.A., Giner J., Futschik M.E. (2016). The Transcriptional Landscape of the Photosynthetic Model Cyanobacterium *Synechocystis* sp. PCC 6803. Sci. Rep..

[27] De Rosa E., Checchetto V., Franchin C., Bergantino E., Berto P., Szabò I., Giacometti G.M., Arrigoni G., Costantini P. (2015). [NiFe]-hydrogenase is essential for cyanobacterium *Synechocystis* sp. PCC 6803 aerobic growth in the dark. Sci. Rep..

[28] Gonçalves C.F., Pacheco C.C., Tamagnini P., Oliveira P. (2018). Identification of inner membrane translocase components of TolC-mediated secretion in the cyanobacterium *Synechocystis* sp. PCC 6803. Environ. Microbiol..

[29] Oliveira P., Martins N.M., Santos M., Pinto F., Büttel Z., Couto N.A.S., Wright P.C., Tamagnini P. (2016). The versatile TolC-like Slr1270 in the cyanobacterium *Synechocystis* sp. PCC 6803. Environ. Microbiol..

[30] Pardo Y.A., Florez C., Baker K.M., Schertzer J.W., Mahler G.J. (2015). Detection of outer membrane vesicles in *Synechocystis* PCC 6803. FEMS Microbiol. Lett..

[31] Prados-Rosales R., Weinrick Brian C., Piqué Daniel G., Jacobs William R., Casadevall A., Rodriguez G.M. (2014). Role for *Myco*-*bacterium tuberculosis* Membrane Vesicles in Iron Acquisition. J. Bacteriol..

[32] Mesnage S., Fontaine T., Mignot T., Delepierre M., Mock M., Fouet A. (2000). Bacterial SLH domain proteins are non-covalently anchored to the cell surface via a conserved mechanism involving wall polysaccharide pyruvylation. EMBO J..

[33] Stanier R.Y., Kunisawa R., Mandel M., Cohen-Bazire G. (1971). Purification and properties of unicellular blue-green algae (order Chroococcales). Bacteriol. Rev..

[34] Letunic I., Bork P. (2018). 20 years of the SMART protein domain annotation resource. Nucleic Acids Res..

[35] Letunic I., Khedkar S., Bork P. (2021). SMART: Recent updates, new developments and status in 2020. Nucleic Acids Res..

[36] Carlone G.M., Thomas M.L., Rumschlag H.S., O Sottnek F. (1986). Rapid microprocedure for isolating detergent-insoluble outer membrane proteins from *Haemophilus* species. J. Clin. Microbiol..

[37] Fisher M.L., Allen R., Luo Y., Curtiss R. (2013). Export of Extracellular Polysaccharides Modulates Adherence of the Cyano-bacterium *Synechocystis*. PLoS ONE.

[38] Heidorn T., Camsund D., Huang H.-H., Lindberg P., Oliveira P., Stensjö K., Lindblad P., Voigt C. (2011). Chapter Twenty-Four -Synthetic Biology in Cyanobacteria: Engineering and Analyzing Novel Functions. Methods in Enzymology.

[39] Oliveira P., Pinto F., Pacheco C.C., Mota R., Tamagnini P. (2015). HesF, an exoprotein required for filament adhesion and aggregation in *Anabaena* sp. PCC 7120. Environ. Microbiol..

