# Supplementary material for: The Role of Outer Membrane Protein(s) Harboring SLH/OprB-Domains in Extracellular Vesicles’ Production in Synechocystis sp. PCC 6803
Source: Plants (Basel). 2021 Dec 14;10(12):2757. doi: 10.3390/plants10122757 (PMC8707739; doi:10.3390/plants10122757)
Supplement: Supplementary file 1 [file plants-10-02757-s001.zip › plants-1472691-supplementary.pdf]

Supplementary Material

Table S1 - Transcript levels analysis of the *Synechocystis* sp. PCC 6803 transcriptional units (TU) harboring genes encoding SLH/OprB-domains outer membrane proteins under 10 different environmental conditions, as determined by [18].

TU ID	TU start	TU end	Strand	5'UTR	genes	15 ºC	42 ºC	$-CO_2$	Dark	-Fe	HL	-N	-P	Exp. p.	Stat. p.	Max reads	Average	Max/Avg	Sense tags
TU971	958084	960116	+	53	1	40362	45295	38272	9363	45113	95667	82402	66762	49195	29046	95667	50147,7	1,91	***slr1841***
TU599	610937	614020	+	120	3	21331	15443	7420	243	42214	53026	11858	43046	22829	4413	53026	22182,3	2,39	***slr1908***, *slr1909*, *slr1910*
TU1141	1116114	1111170	-	93	4	1678	0	58	0	214	259	2823	0	13761	180	13761	1897,3	7,25	*sll1270*, ***sll1271***, *ssl2507*, *sll1272*
																			sll0772
TU3332	3168888	3176441	+	166	5	30343	1201	345174	2673	5711	1005917	5985	26025	207607	180	1005917	163081,6	6,17	*slr0040*, *slr0041*, ***slr0042***, *slr0043*, *slr0044*
TU3630	3456162	3454008	-	106	1	0	33	19	0	1382	199	75	39	210	45	1382	200,2	6,9	***sll1550***

In bold are highlighted the genes encoding SLH/OprB-domains outer membrane proteins studied in this work.

Table S2. Oligonucleotides used in this work.

Oligo	Application	Sequence (5’ to 3’)	Product size (bp)	Restriction Enzyme	T_a (ºC)
slr1841 5'F	slr1841 5' recombination plaform	Aaa**ctcgag**agtccctgttagtgagccct	651	XhoI	63
slr1841 5'R		Aaactgcagcgagacgagtgaccaacagg		PstI	
slr1841 3'F	slr1841 3' recombination plaftorm	Aaactgcagtgggttacttggctagcacg	663	PstI	63
slr1841 3'R		Aaa*ggatcc*gcagagggttagcagtggtt		BamHI	
slr1908 5'F	slr1908 5' recombination platform	Aaa**ctcgag**agtccacaggtgaattggcc	580	XhoI	63
slr1908 5'R		Aaactgcaggcgccgaagttgtcaatgtt		PstI	
slr1908 3'F	slr1908 3' recombination platform	Aaactgcagctggattggcggtactggtt	668	PstI	63
slr1908 3'R		Aaa*ggatcc*agggcatcaaaccaccactc		BamHI	
slr0042 5'F	slr0042 5' recombination platform	Aaa**ctcgag**gtgacatcggtttcggagtt	647	XhoI	63
slr0042 5'R		Aaactgcagggttggagattttggggatt		PstI	
slr0042 3'F	slr0042 3' recombination platform	Aaactgcaggcctaacgatgtggtcgatt	629	BamHI	63
slr0042 3'R		Aaa*ggatcc*tagagtcgccttccggatta		PstI	
sll1550 5'F	sll1550 5' recombination platform	Aaa**ctcgag**gggacagatcacttccgtgt	566	XhoI	63
sll1550 5'R		Aaactgcagacactggctggaaagtaccg		PstI	
sll1550 3'F	sll1550 3' recombination platform	Aaactgcagacccgtagcccgatctattt	581	PstI	63
sll1550 3'R		Aaa*ggatcc*tcaatgtcagtggtggcaat		BamHI	
sll0772 5'F	sll0772 5' recombination platform	Aaa**ctcgag**ggcaatggcgttaatcaagt	689	XhoI	63
sll0772 5'R		Aaactgcagacagtacccaagcgcagagt		PstI	
sll0772 3'F	sll0772 3' recombination platform	Aaactgcagagctggctttaattggcaga	636	PstI	63
sll0772 3'R		Aaa*ggatcc*ttaagcacgacccaaaaacc		BamHI	
sll1271 5'F	sll1271 5' recombination platform	Aaa**ctcgag**aacgacaatgccaaaacctc	648	XhoI	63
sll1271 5'R		Aaactgcagggcggcaaagttaaaatcag		PstI	
sll1271 3'F	sll1271 3' recombination platform	Aaactgcagatcgcccccatttatttacc	574	PstI	63
sll1271 3'R		Aaa*ggatcc*cggtggtagcgggagtaata		BamHI	
pUC 4K F	Kanamycin resistance cassette	aaactgcagtgaggtctgcctcgtgaagaa	1226	PstI	64
pUC 4K R		aaactgcagaaagccacgttgtgtctcaaa		PstI	

The pair of oligonucleotide used for the amplification of the kanamycin resistance cassette was taken from [29].

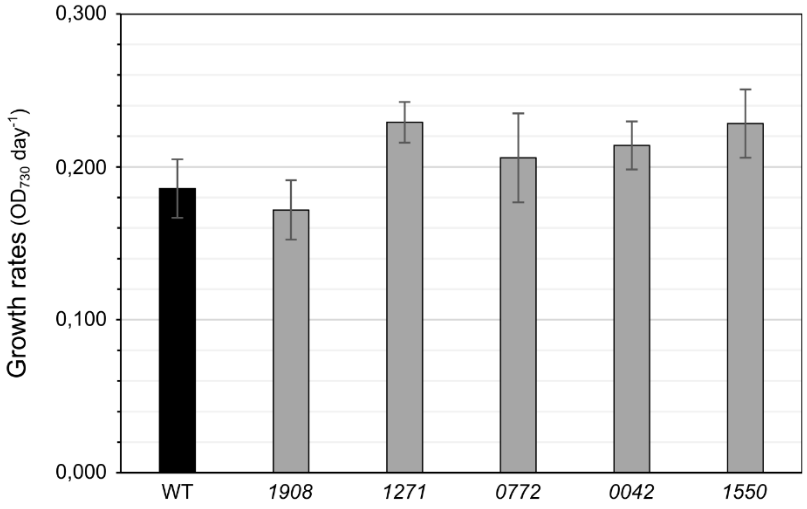

Figure S1. *Synechocystis* sp. PCC 6803 disruption mutants' growth rates determined in cultures grown under standard conditions. *Synechocystis* cultures of the wild-type (WT) and mutant strains *slr1908* (*1908*), *slr1271* (*1271*), *sll0772* (*0772*), *slr0042* (*0042*) and *sll1550* (*1550*) were grown in BG11 liquid medium, in 96-well clear bottomed microplates at 30 ºC with a 16 h light (50 µmol photons m^{-2} s^{-1})/ 8 h dark cycle regime, with agitation, for 4 days. Every day, cell cultures were suspended and their optical density (OD_{730}) measured on a microplate reader. Growth rates were determined between the first and fourth day of cultivation. Error bars represent the standard deviation of three independent biological replicates.
